# Discovery and Functional Analysis of Secondary Hair Follicle miRNAs during Annual Cashmere Growth

**DOI:** 10.3390/ijms24021063

**Published:** 2023-01-05

**Authors:** Minglin Wang, Han Dai, Shengda Sheng, Yanlei Liu, Shuyi Zhang, Wenlin Bai, Huiling Xue

**Affiliations:** College of Animal Science and Veterinary Medicine, Shenyang Agricultural University (SYAU), Shenyang 110866, China

**Keywords:** miRNA, secondary hair follicle, cashmere, hair growth regulation, phase transition

## Abstract

Secondary hair follicles (SHFs) produce the thermoregulatory cashmere of goats. MicroRNAs (miRNAs) play indispensable roles in hair follicle formation and growth. However, most studies examining miRNAs related to cashmere have been performed on goat skin. It remains unclear which miRNAs are highly expressed in SHFs or how miRNAs affect cashmere growth. In the present study, we isolated the SHFs under a dissecting microscope and analyzed the miRNA signatures during annual cashmere growth. Small-RNA sequencing followed by genome-wide expression analysis revealed that early anagen is a crucial phase for miRNA regulation of the cashmere growth, as revealed by two predominant groups of miRNAs. Although they exhibited opposite expression patterns, both groups demonstrated sharp changes of expression when in transit from early anagen to mid-anagen. In addition, we identified 96 miRNA signatures that were differentially expressed between different phases among 376 miRNAs. Functional analysis of the predicted target genes of highly expressed or differentially expressed miRNAs indicated that these miRNAs were involved in signal pathways associated with SHF development, regeneration, and regression. Furthermore, miR-143-3p was preferentially expressed in SHFs and *Itga6* was identified as one of targets. The dual-luciferase and in situ hybridization assay demonstrated that miR-143-3p directly repressed the expression of *Itga6*, suggesting a possible novel role for miR-143-3p in cashmere growth.

## 1. Introduction

Hair is a primary characteristic of mammals, and it is produced by hair follicles (HFs) anchored in the subcutis. There are two types of hair follicle in cashmere goats, the primary HFs (PHFs) that create the protective coarse hair, and the secondary HFs (SHFs) that produce the thermoregulatory cashmere, which is a special soft hair fiber of great resilience with high economic value because of its heat-preservation feature. As a type of smaller size HF, SHFs usually surround the PHF in a follicle group [1]. Mature hair follicles periodically regenerate, undergoing repetitive cycles of active growth (anagen), apoptosis-driven regression (catagen), and relative rest (telogen). In contrast to asynchronous growth with a long cycle, such as human hair and goat wool, cashmere hair follicles (SHFs) show synchronous annual growth, which is shed every May for Liaoning cashmere goats. Many signal pathways are implicated in controlling HF growth, such as Wnt, Hedgehog, Notch, TGF-β/BMP, FGF (fibroblast growth factor), and TNF [2,3,4,5]. Numerous signals have been discovered in HF formation and growth, including transcription factors, growth factors, cytokines, neuropeptides, and hormones, such as SOX9 (sex-determining region Y-box 9), FGF7 (fibroblast growth factor 7), NFI (nuclear factor I), TRH (thyrotropin-releasing hormone), and corticosterone [6,7,8,9,10]. However, several findings have shown that the growth and development of primary and secondary HFs are regulated by distinctive signal pathways, for example, they respond differentially to EDA signaling [11,12,13,14]. PHFs are not formed in *Eda* mutant Tabby mice, but SHFs developed normally [13,14,15,16,17]. Melatonin was found to promote the initiation and maturation of secondary follicles and increasing their population, while having little effect on PHF population [1]. Therefore, understanding the molecular mechanisms controlling the growth and development of SHFs, rather than skin or PHFs, might improve cashmere quality and increase yield.

MicroRNAs (miRNAs) are a class of small noncoding RNAs that typically repress expression of proteins by inducing degradation or translational inhibition of their target mRNAs [18,19]. Increasing evidence suggests that microRNAs play crucial roles in HF growth, regeneration, and regression. Of the specific miRNAs identified as crucial modulators, miR-24 affects hair follicle morphogenesis by targeting Tcf-3 and controls the regenerative competence of hair follicle progenitors by targeting Plk3 [20]. miR-29a/b1 inhibits the lineage progression of hair follicle stem cells [21]. miR-125b represses the differentiation of hair follicle stem cells [22]. The miRNA-200 family, although preferentially expressed in the epidermis, is reported to regulate cell adhesion and proliferation in hair morphogenesis [23]. miR-22 regulates HF regression and keratinocyte progenitor cell differentiation [24]. In addition, miRNAs are important in maintaining the ability of adult HFs in normal cycles of growth [25,26]. A few miRNAs were reported involved in regulating cashmere or wool growth. miR-30b and miR-143 regulate the proliferation of dermal papilla cells in Shanbei white cashmere goats and Hu sheep, respectively [27,28].

Most studies on miRNAs related to cashmere have been performed on skin, which is composed of the epidermis, dermis, and hypodermis. However, hair follicles and epidermis differentially express discrete miRNAs. For example, the miR-200 family and miR-19/miR-20 family were expressed preferentially in the epidermis. In contrast, the miR-199 family encompassing miR-199a and miR-199b was exclusively expressed in the hair follicles [29]. In the present study, we explored miRNAs and their functional significance in cashmere follicles (SHFs) from Liaoning cashmere goats. We isolated SHFs under the microscope by cutting out the epidermis and removing the connective tissues. The secondary hair follicles were smaller in size and grew in a follicle group, which could be clearly distinguished from single units of primary hair follicles. We performed small RNA sequencing experiments on the collected SHFs to discover miRNAs that were highly or preferentially expressed in SHFs.

To explore the role of miRNAs in cashmere growth, we identified the differentially expressed (DE) miRNAs between the consecutive phases. The potential functions of the highly expressed or DE miRNAs were further analyzed by examining their target genes. In addition, we studied the function of miR-143-3p, which would help to illustrate the regulatory mechanism of miRNA on cashmere growth.

## 2. Results

### 2.1. Identification of miRNAs in Secondary Hair Follicles

**Figure 1:**
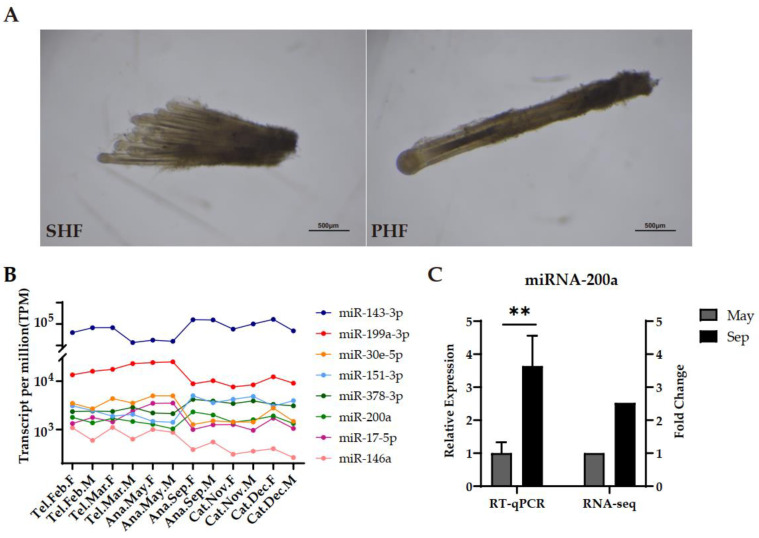
Two types of hair follicle and representative miRNAs identified in SHFs from Liaoning cashmere goats. (**A**) The SHF group (left) and PHF (right) isolated in vitro from the back skin of Liaoning cashmere goat. (**B**) Expression patterns of representative miRNAs. TPM, Transcripts Per Million; Ana, anagen; Cat, catagen; Tel, telogen; F, female; M, male. (**C**) Relative expression of miR-200a in May and September detected by RT-qPCR (left *y*-axis). Fold change is the ratio of mean TPM in September to that in May in sequencing data (right *y*-axis). Data are means ± SD for all bar graphs. *n* = 3 for bar groups. ** *p* < 0.01. Significant analysis was performed by one-sided Student’s *t* test.

To systematically discover miRNA transcripts with potential biological roles during annual cashmere growth, we isolated SHF clusters, which manifested a different morphology from the PHFs (Figure 1A). After a great number of SHFs were collected, we performed small RNA sequencing experiments across six time points, each with two biological replicates (male and female), representing phases of anagen (May and September), catagen (November and December), and telogen (February and March). Around 4.7 × 10^6^ reads for each sample were obtained, with 5.65 × 10^7^ reads in total. More than 92% of the reads of all samples were mapped to the reference genome (CHIR_2.0). The bioinformatics software miREvo (v.1.1) [30] and mirDeep2 (v.2.0.0.5) [31] were used to predict the potential miRNA candidates. After comparison to the miRbase goat database, a total of 376 miRNAs were identified, including 338 published miRNAs and 38 novel candidates (Figure S1 and Table S1). Among them, a subset of 23 miRNAs dominated the total miRNA expression, showing extremely high expression throughout all phases (TPM > 5000). These included miR-26a, miR-143, miR-148a, the let-7 family, and the miR-199 family, especially it contained the novel candidate miR-203 with an average TPM > 40,000. A total of 34 miRNAs exhibited relatively higher expression (1000 < TPM < 5000), and others expressed TPM values lower than 1000 (Figure 1B). Raw RNA-seq data files and TPM (transcripts per million clean tags) values were submitted to the Gene Expression Omnibus (GEO) database with accession no. GSE220211. Real-time PCR verified the accuracy and reliability of our sequencing data. As expected, miR-200a expression was higher in September than in May (Figure 1C). In addition, it showed dynamic changes similar to RNA-seq expression patterns (Figure 1C).

To reveal the potential biological roles of the expressed miRNAs in SHFs, we predicated the targets of each miRNA using the programs miRanda (v.3.3) and TargetScan (v.7.0). To further confirm the relationship between each miRNA and its potential targets, we evaluated the correlation for each relationship pair. The Pearson correlation coefficient (PCC) between the two serials of the expression levels of the miRNA-target pair throughout the hair growth cycle was used to measure the similarity of expression patterns. We used PCC values of 0.4 and −0.4 as cutoffs for positive and negative correlations, respectively. These cutoffs were established in previous studies. After this filtering, only the strongly correlated relationships were retained for further study.

### 2.2. Prominent miRNA Signatures during the Transition from Early Anangen to Mid-Anagen

To uncover the specific expression patterns of miRNAs among three consecutive phases, we assayed miRNAs that differentially expressed with potential roles in the phase transition during the hair cycle. Given the appreciable difference in gene expression profiles between May and September, we compared May and September to other phases. We defined the molecular signatures as miRNAs with expressions 2-fold higher or lower in each phase relative to other phases and an FDR less than 0.05. Following these selection criteria, we discovered 96 differentially expressed miRNAs in total over the whole hair cycle, including 89 published miRNAs and 7 novel miRNA candidates.

**Figure 2:**
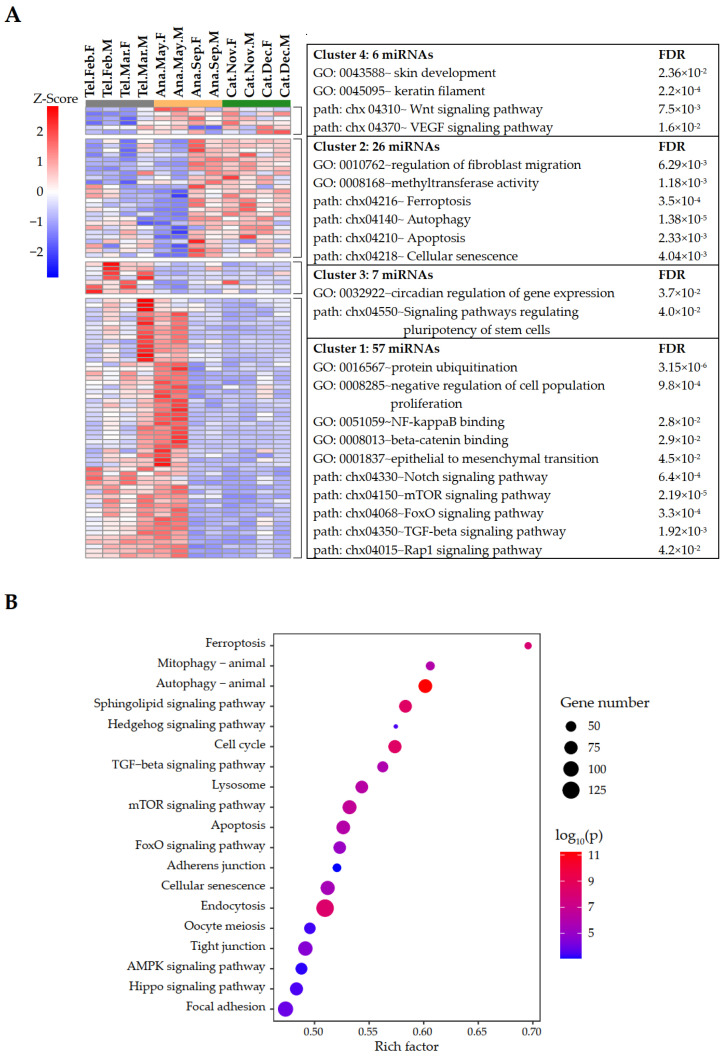
Differentially expressed miRNAs between different phases. (**A**) Hierarchical clustering of differentially expressed miRNAs and functional analysis of their targeted genes. Gene ontology (GO) and KEGG analysis of the targeted genes of DE miRNAs. Ana, anagen; Cat, catagen; Tel, telogen; F, female; M, male; FDR, false discovery rate. (**B**) KEGG pathway enrichment analysis of Cluster 2 miRNA target genes.

Hierarchical clustering revealed that these differentially expressed miRNAs (DE miRNAs) could be divided into four clusters, each representing a unique set of expression patterns, reflective of the dynamic properties of phase transition (Figure 2A). Surprisingly, this revealed that two predominant clusters (Cluster 1 and Cluster 2), accounting for about ~86% of the DE miRNAs, demonstrated a sharp change of expression when in transit from early anagen (May) to mid-anagen (September). This was surprising because the two anagen subperiods shared more mRNA transcriptional similarities than telogen and catagen, while the expression patterns of miRNA signatures were markedly distinctive. The expression of miRNAs in the largest cluster (Cluster 1) progressively rose in telogen and peaked in early anagen (May), while markedly decreased in the following phases (anagen and catagen). These included miR-199a-3p, miR-30e-5p, miR-17-5p, and miR-146a, whose targets were functionally enriched in pathways and biological processes related to hair development and growth, including Notch, mTOR, FoxO, TGF-beta, and Rap1 signaling pathways, beta-catenin binding, and epithelial-to-mesenchymal transition (Figure 2A). All enriched KEGG pathways are shown in Figure S2A. These signatures manifested a series of active signaling processes that are important in HF development. The discovery that miRNA expression peaked during the initiation of a new cycle of hair growth suggested that these miRNAs may play an important role in promoting cell differentiation. In contrast, another major cluster (Cluster 2) contained miRNAs with enhanced expression in anagen (September) and sustained comparable expression in catagen, while their expression reached the lowest level in early anagen (May). This cluster included miR-151-3p, miR-378-3p, miR-143-3p, and miR-200a, which may regulate the transition from active growth to regression. GO and KEGG analysis of their target genes found them related to cell death, such as ferroptosis, autophagy, apoptosis, cellular senescence, and signaling pathways, such as Hedgehog, TGF-beta, mTOR, and Hippo (Figure 2B and Supplementary File S1). Notably, ~70% of the genes in the ferroptosis pathway were their potential targets, whose expression strongly correlated with those of Cluster 2 miRNAs (Figure 2B). Recently, ferroptosis has been increasingly reported in multiple pathological conditions, but there are few reports on ferroptosis under physiological conditions such as HF growth or regression, in which apoptosis (another well-known regulated cell death form) was mostly studied [32,33,34]. Although these two predominant clusters showed opposite dynamic expression patterns, both demonstrated a sharp change of expression when in transit from early anagen to mid-anagen, compared to telogen−early anagen or anagen−catagen transitions. These results indicated the concordant expression changes of miRNA signatures, functioning together to orchestrate the annual cashmere growth. Cluster 3 was a small cluster of seven miRNAs identified as telogen-specific signatures with specifically high expression throughout telogen, including miR-9-5p and miR-29b-3p. It was observed in previous studies that miR-29a/b1 deficiency in mice accelerates hair follicle stem cell (HFSC) lineage progression in telogen. Conversely, the sustained miR-29a/b1 overexpression in telogen causes hair loss by inhibiting the proliferation of HFSCs and matrix cells and preventing their differentiation [21]. Telogen-specific miRNA signatures may function in maintaining SHF homeostasis in a relatively dormant resting state by targeting genes involved in pathways regulating the pluripotency of stem cells, cell cycle, VEGF, and ErbB signaling (Figure S2B and Supplementary File S1). The expression of miRNAs such as miR-423-5p and miR-345-3p in Cluster 4 was low during telogen and high throughout anagen and catagen. GO and KEGG analysis of target genes was found to be related to HF growth such as keratin filament, skin development, and Wnt and VEGF signaling pathways (Figure S2C and Supplementary File S1). Taken together, these identified miRNA signatures extended previous findings, and our results confirmed the markedly important roles of these miRNAs in cyclic cashmere growth and provided new insights into previously unexplored biological processes, thereby offering valuable starting points for future work.

### 2.3. Molecular Characteristics of Highly Expressed miRNAs in SHFs

**Figure 3:**
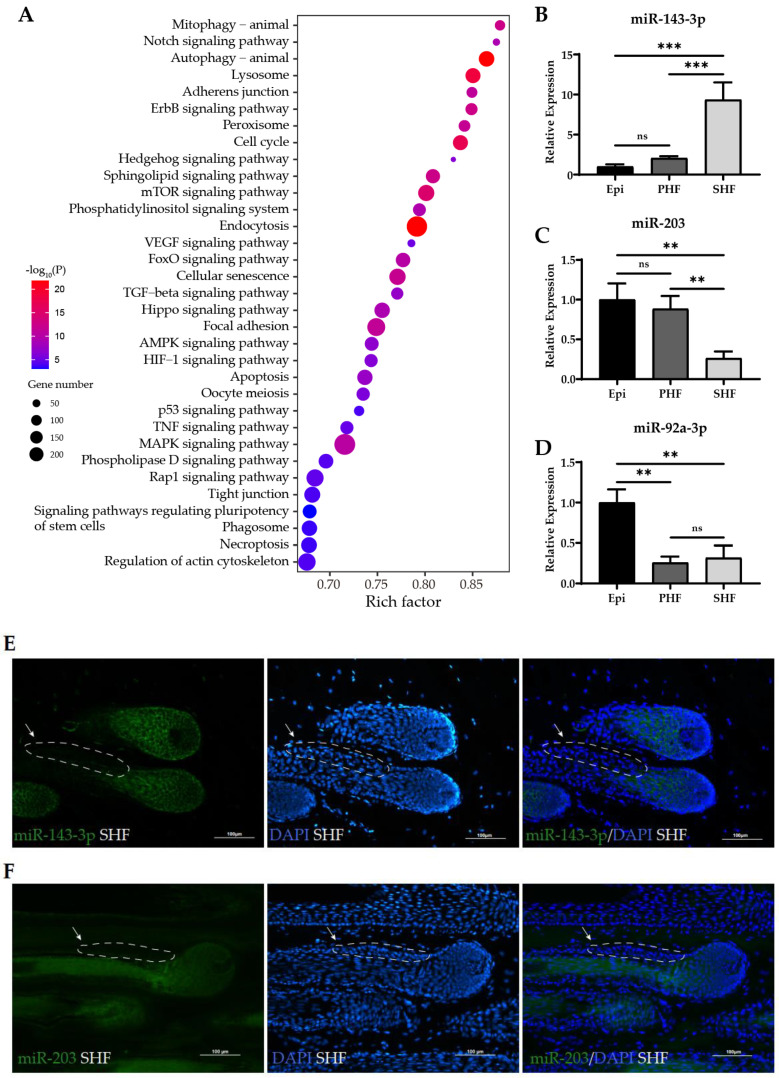
Functional analysis of highly expressed miRNAs in SHFs and differential expression of selected miRNAs. (**A**) KEGG pathway enrichment analysis of target genes of highly expressed miRNAs (TPM ≥ 500 in at least two samples). Rich factor indicates the ratio of target genes enriched in the pathway to genes annotated in the pathway. (**B**–**D**) Relative expression in the epidermis, PHFs, and SHFs of miR-143-3p (**B**), miR-203 (**C**), and miR-92a-3p (**D**) by RT-qPCR. *n* = 3 for all groups. Data are means ± SD for all bar graphs. Significant analysis was performed by one-sided Student’s *t* test. ns, not significant; ** *p* < 0.01; *** *p* < 0.001. Epi, epidermis; PHF, primary hair follicle; SHF, secondary hair follicle. (**E**,**F**) Fluorescence in situ hybridization (FISH) assays of miR-143-3p (**E**) and miR-203 (**F**) in SHFs during anagen. The green signals represent the expression of miR-143-3p or miR-203. All nuclei were stained with DAPI (blue). White arrowheads indicate the bulge region outlined by dashed line. Scale bars, 100 μm.

Numerous miRNAs are highly expressed throughout the cashmere growth cycle, indicative of their essential roles in SHF formation and growth, regardless of whether they are differentially expressed between two different phases. To evaluate the overall impact of miRNAs on cashmere growth, we explored the highly expressed miRNAs with TPM ≥ 500 in at least two samples and their functional significance with enrichment analysis on their potential targets. All 83 abundant miRNAs expressed in SHFs are listed in Supplementary File S2. Subsequent GO and KEGG analysis showed that the highly expressed miRNAs were enriched in many signal pathways or processes related to HF growth and development, and epidermis development, such as TGF-beta, Notch, Hippo, Hedgehog, VEGF, Rap1, mTOR, and ErbB signaling pathways, and pathways regulating the pluripotency of stem cells, cellular response to epidermal growth factor stimulus, regulation of epithelial-to-mesenchymal transition, and canonical Wnt signaling pathway (Figure 3A and Supplementary File S1).

To determine whether the miRNAs were preferentially expressed in SHFs, we performed RT-qPCR for a selected set of miRNAs in SHFs, PHFs, and the epidermis isolated from cashmere goat skin (Figure 3B–D). miR-143-3p was notable for its strong enrichment in SHFs. Its expression was about 10 times greater in SHFs than in the epidermis or in PHFs (Figure 3B). Conversely, the expression level of miR-203 in SHFs was only 1/8th that in the epidermis or PHFs (Figure 3C). Specific to the epidermis was miR-92a-3p, which was more abundant in the epidermis than in the hair follicles (Figure 3D). The results revealed the distinctive expression patterns of miRNAs in SHFs, PHFs, and the epidermis.

To investigate the expressed localization of miRNAs in hair follicles, we performed an RNA fluorescence in situ hybridization (FISH) assay for miR-143-3p and miR-203, both of which are particularly abundant in hair follicles. A strong miR-143-3p signal was evident in the matrix of SHFs (Figure 3E) and PHFs (Figure S3), whereas no signal was detected in the bulge. The results demonstrated that miR-143-3p was expressed in transit-amplifying matrix cells, but not expressed in pluripotent stem cells (Figure 3E), indicating a potential function in hair follicle stem cell progeny. A specific miR-203 activity was detected in the hair shaft (Figure 3F), which may support previous observations that downregulated miR-203 induces the inhibition of hair shaft growth [35], indicating that miR-203 may be involved in the terminal differentiation of cashmere follicles.

### 2.4. MiR-143-3p Directly Represses the Expression of Itga6

**Figure 4:**
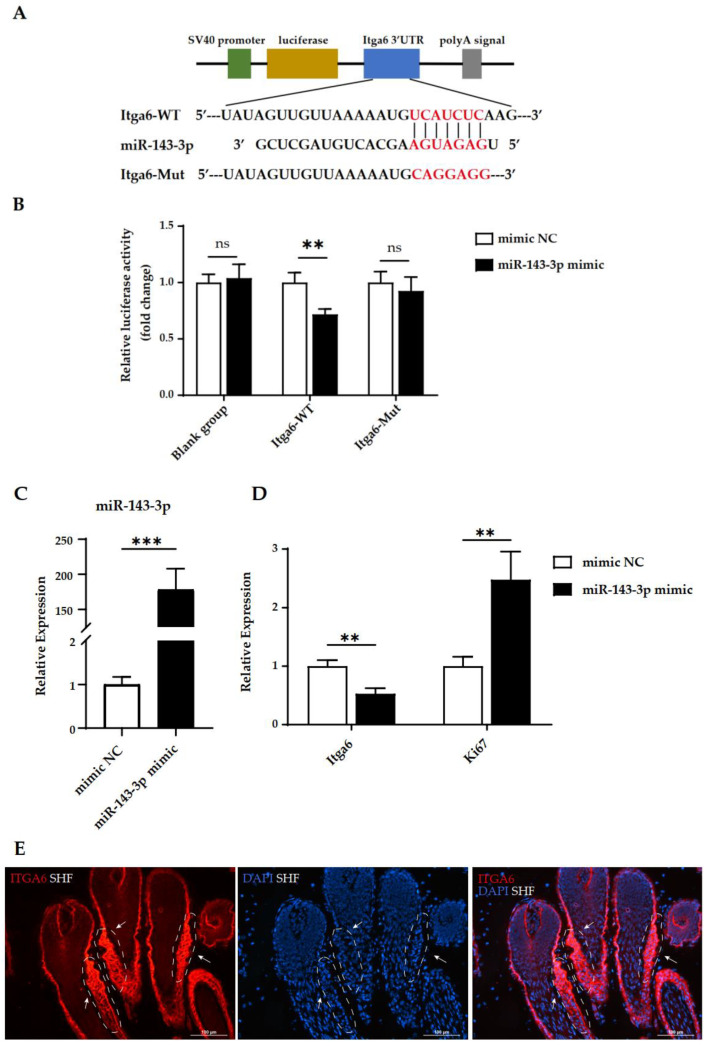
miR-143-3p directly represses the expression of *Itga6* in SHFs. (**A**) The wild-type or mutant *Itga6* 3′UTRs determine the miR-143-3p binding site. (**B**) The relative luciferase activity of the wild-type or mutant *Itga6* 3′UTR in 293T cells after transfections with the miR-143-3p mimic and corresponding control. (**C**) The expression of miR-143-3p after a transfection with miR-143-3p mimic, negative control (NC). (**D**) Relative expression of *Itga6* and *Ki67* after the overexpression of miR-143-3p in secondary HFSCs. *n* = 3 for all groups. Significant analysis was performed by one-sided Student’s *t* test. Data are means ± SD for all bar graphs. ** *p* < 0.01, *** *p* < 0.001, ns, not significant. (**E**) Expression of ITGA6 in SHFs was examined by immunofluorescence. The red signals represent ITGA6 staining. The nuclei were stained with DAPI (blue). White arrowheads indicate the bulge region outlined by white dashed lines. Scale bars, 100 μm.

As miR-143-3p is particularly more abundant in SHFs than in the epidermis and PHFs in cashmere goats, it was selected for further analysis. Two programs (TargetScan and miRanda) were used to predict the target sites of miR-143-3p (Figure 4A). *Itga6* (integrin subunit alpha 6) was one of the predicted targets. *Itga6* is an essential marker gene for pluripotent cells of the hair follicle, which influences the balance between stem cell renewal and differentiation [36] and is targeted by miR-143-3p in human gallbladder carcinoma to suppresses tumor growth and angiogenesis [37]. The 5354–5360 region of the *Itga6* 3′ UTR contains a predicted miR-143-3p binding site (Figure 4A). To prove the direct binding of miR-143-3p on the 3′ UTR regions of the *Itga6* mRNA, we constructed a dual-luciferase reporter plasmid containing a fragment of the *Itga6* 3′ UTR across the miR-143-3p binding sites (Figure 4B). The dual-luciferase reporters were co-transfected with miR-143-3p mimics or control mimics (NC mimics) into the 293T cells. The dual-luciferase activity assays showed a significant reduction of *Itga6* expression by the miR-143-3p mimics. Conversely, the expression of *Itga6* was not affected when the miR-143-3p target site was mutated (Figure 4B). In addition, the endogenous expression of *Itga6* was significantly decreased in HFSCs with overexpressed miR-143-3p compared with the miRNA-NC group (Figure 4C,D). Moreover, overexpression of miR-143-3p significantly upregulated the expression of proliferation marker *Ki67* (Figure 4D). These results indicated that the effect of miR-143-3p on *Itga6* is direct and is mediated by a specific 3′ UTR target site in the secondary hair follicles of cashmere goats. miR-143-3p can enhance the proliferation of HFSCs or hair follicle stem cell progeny to regulate the SHF growth.

We next localized the expression of ITGA6 by immunofluorescent staining. In the anagen SHFs, high ITGA6 activity was restricted to the bulge and outer root sheath (ORS), but there was only a faint signal in the matrix region where miR-143-3p was abundant (Figure 3E and 4E). The discovery that ITGA6 and miR-143-3p were expressed in an opposite manner spatially suggested the negative regulation of ITGA6 by miR-143-3p. The localization results indicated that miR-143-3p may have an important effect on the hair follicle stem cell progeny in the hair matrix by repressing the expression of ITGA6. Taken together, our studies provided evidence that miR-143-3p acts by targeting and negatively regulating the expression of *Itga6*, thereby affecting the cell renewal and differentiation during cashmere growth.

### 2.5. Regulatory Network of miR-143-3p in SHF

**Figure 5:**
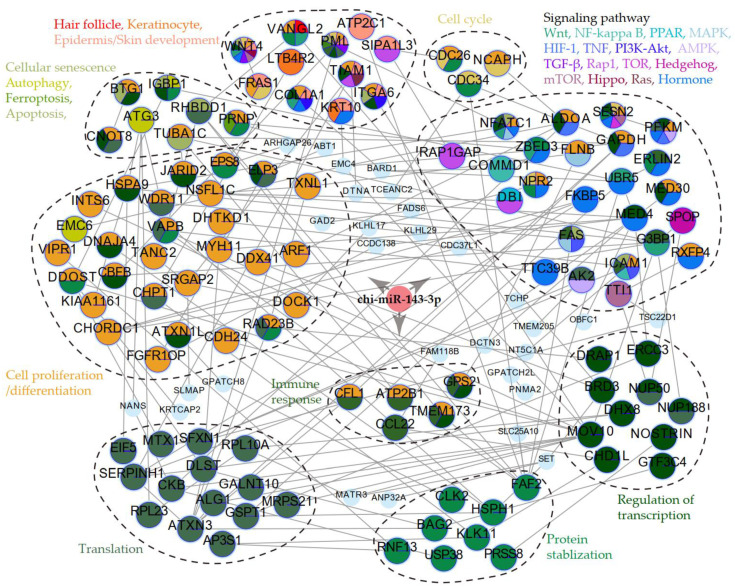
Regulatory network of miR-143-3p and its target genes. Colors represent different biological functions. The interactions between miR-143-3p and its target genes are indicated by three arrowheads in bold gray.

To explore the potential roles and molecular mechanisms of miR-143-3p in regulating the SHF growth and/or regression, a regulatory network between miR-143-3p and its potential targets was constructed (Figure 5). In this network, each pair of miR-143-3p and target mRNA had similar expression profiles or opposite expression profiles determined by Pearson correlation coefficient (PCC) as correlated or anti-correlated interaction. Interactions between target genes were elicited from the BioGRID database (http://thebiogrid.org/, accessed on 25 May 2022) and filtered by PCC. According to the annotated functions, the target genes could be categorized into several groups, including signaling pathways or factors related to hair follicle morphogenesis and development, cell proliferation and differentiation, transcription and protein regulation, and various cell death programs (Figure 5). These findings provided additional insights into how miR-143-3p regulates SHF regeneration or regression in cashmere goats.

## 3. Discussion

In the current study, through systematic analysis of miRNAs from isolated secondary hair follicles, we identified and characterized differentially expressed or consistently highly expressed miRNAs across the annual growth cycle of cashmere and demonstrated their differential expression features and importance in cashmere regeneration and shedding. Most studies examining cashmere follicle miRNAs have been performed on goat skin, which is composed of multiple different cell types and accessory organs (HFs and sweat glands). Moreover, there are two types of HFs in goat skin—SHFs and PHFs. Compared with the synchronized seasonal growth of SHFs, the growth and apoptosis of PHFs are unsynchronous and occur throughout the year, regardless of the seasonal changes. To eliminate the influence of the proportions of the different cell types in the skin sample and/or of genes differentially expressed in a specific cell type, we analyzed the isolated SHFs instead of the skin samples; miRNAs characterized here were more specifically expressed in SHFs, and we largely avoided those from other cell types in the skin samples, especially removing the noise signatures of the phase transition from PHFs. Therefore, the functional analysis of predicted target genes clearly revealed the abundance of signaling pathways involved in the HF growth.

It was intriguing to find that early anagen is a markedly crucial phase of the miRNA signatures suggested by two predominant groups of miRNAs that exhibited completely opposite dynamic patterns, but both of which demonstrated sharp changes of expression when in transit from early anagen to mid-anagen. In addition, the expression of these miRNA either peaked or bottomed out in early anagen throughout the annual cashmere growth cycle. Although the special patterns were discovered in early anagen, since the earlier time point (April) was not examined, it was possible that the predominant miRNAs exhibited the highest or lowest expression and striking changes in late telogen/early anagen. The concordant expression changes of these miRNA signatures to function together further confirmed the markedly important roles of these miRNA in regulating the phase transition and cyclic growth of cashmere.

Our results indicated that miR-143-3p was preferentially expressed in SHFs compared to the epidermis and PHFs. Analogous to the observation in Hu sheep, miR-143 was highly expressed in small-wave wool skin with the best lambskin quality, compared with large-wave wool skin [38]. In our study, miR-143-3p was in Cluster 2, which showed enhanced expression in anagen and catagen, while showing progressively reduced expression in telogen until reaching the lowest point in early anagen (May). This was consistent with and extended the previous observations of Shanbei white cashmere goats, where miR-143 was highly expressed in the hair follicles during anagen and catagen [39]. Our RNA in situ hybridization results demonstrated that miR-143-3p is localized in the hair matrix, but not in the bulge. *Itga6*, a target of miR-143-3p, which was validated by dual luciferase assay, showed spatially differential expression with high activity in the bulge and outer root sheath, but a weak signal in the hair matrix. These results indicated a possible novel role for miR-143-3p in modulating SHF growth through negatively regulating ITGA6 expression.

In summary, we discovered and characterized miRNAs from isolated SHFs and demonstrated that miR-143-3p was preferentially expressed in SHFs and may regulate the cashmere growth by repressing *Itga6* expression in Liaoning cashmere goats. We therefore propose the miR-143-3p–*Itga6* axis as a novel pathway likely regulating the proliferation of stem cell progeny and/or HFSC lineage progression. As future studies are conducted, it will be interesting to explore whether the intervening miR-143-3p expression affects the cashmere quantity and/or quality.

## 4. Materials and Methods

### 4.1. Experimental Animals

All experimental cashmere goats were bred in the Modern Agricultural Production Base Construction Engineering Center of Liaoning Province, Liaoyang. Three male and three female cashmere goats, all 1.5 years old, were selected in each time point (February, March, May, September, November, and December). A piece of back skin (0.5 × 0.5 cm) was acquired from each animal and brought to laboratory in precooled saline. The secondary hair follicles were isolated by cutting out the epidermis and removing the connective tissues, and they were separated from the primary hair follicles under a dissecting microscope (SMZ150, Motic, Xiamen, China). The separated clean SHF clusters were temporarily immersed in precooled saline placed on ice and collected every 10 min.

### 4.2. Small RNA Library Preparation and Small RNA Sequencing

Total RNA was extracted from these secondary follicles using RNAiso Plus as per the manufacturer’s instructions (Takara, Dalian, China). A 1% agarose gel assay confirmed the lack of decomposition and contamination of the extracted RNA. The quality of total RNA was detected by Nanodrop (detected OD 260/280) (Thermo Fisher, San Francisco, CA, USA), Agilent 2100 (Agilent Technologies, Santa Clara, CA, USA), and electrophoresis. RIN values exceeded 8.5. A total of 3 µg RNA per sample was used as the basis for small RNA library construction by the sequencing company (Novogene, Beijing, China). Finally, the small RNA library was sequenced on the Illumina HiSeq 2500 platform (Illumina, San Diego, CA, USA).

### 4.3. Sequencing Analysis

Clean data were obtained after trimming adapter sequences from the raw sequence reads and removing reads with unknown “N” bases. Filtered reads were mapped to the reference genome (CHIR_2.0) [24]. The abundances of the miRNA sequencing reads were reported in TPM using the Bowtie (v.0.12.9) pipeline to evaluate the expression level of miRNAs. Differentially expressed miRNAs between the consecutive phases were identified using the edgeR package (v.3.38.4) as were those showing 2-fold or more changes and a false discovery ratio (FDR) of <0.05 with the expression greater than 5 counts per million reads in at least two samples

### 4.4. Clustering Analysis, and Enrichment Analysis

Hierarchical clustering was performed using the pheatmap package (v.1.0.12) in R based on the TPM data for DE miRNA genes. Enriched GO (biological process) and KEGG pathway (cellular processes and environmental information processing) analyses were performed using the R module of clusterProfiler (v.4.4.4). An adjusted *p* value < 0.05 was considered statistically significant. A regulatory network was constructed using Cytoscape (v.3.8.2) software to illustrate the relationships between miRNAs and their target genes [40].

### 4.5. Quantitation of Selected miRNA by RT-qPCR

Total RNA of the secondary follicles in telogen and anagen were extracted by RNAiso Plus (Takara, Dalian, China). RNA quality was detected by RNA electrophoresis and measuring the OD 260/280 of RNA. Customized primers for reverse transcription were designed according to the stem-loop method [41]. The reserve transcription kit was used according to instructions (Takara, Dalian, China). The U6 gene was used as the reference gene. The real-time qPCR was performed by a fluorescent quantitation instrument for polymerase chain reaction (Thermo Fisher, San Francisco, CA, USA). The relative expression changes were calculated by the 2^−ΔΔCt^ method. All the primers used for RT-qPCR are listed in Table S2.

### 4.6. In Situ Hybridization

Freshly collected skin samples were quickly frozen with liquid nitrogen. The frozen skin samples were embedded in OCT medium (REF4583, Sakura, Torrance, CA, USA). Samples were sectioned into 8 μm slices using a freezing microtome machine (CM1860, Leica, Shanghai, China). Locked nucleic acid (LNA) probes were compounded in GeneBio Biotech Co., Ltd. (Shanghai, China). The miR-143-3p probe sequence: CGAGCTACAGTGCTTCATCTCA. The miR-203 probe sequence: CTAGTGGTCCTAAACATTTCAC. The in situ hybridization processes followed the published protocol [42].

### 4.7. Immunofluorescence Staining of Skin Tissues

Frozen sections were used for immunofluorescence staining. Sections were air-dried for 20 min and fixed in 4% paraformaldehyde (Servicebio, Wuhan, China) for 10 min. They were blocked with non-specific staining by incubating with 10% donkey serum (Servicebio, Wuhan, China) in PBS-T (1XPBS with 0.2% Triton X-100) for 2 h at room temperature. We added ITGA6 monoclonal antibody (CAT: MA5-16884, Thermo Fisher, Waltham, MA, USA) diluted in PBST (1XPBS with 0.05% TritonX-100, 1:200) with 1% BSA and incubated overnight at 4 °C. A negative control used the incubation buffer without the primary antibody. Samples were then incubated with Alex Fluor 594 conjugated donkey anti-rat IgG (CAT: A-21209, Thermo Fisher, Waltham, MA, USA) for 1 h in the dark. Slides were counterstained by DAPI and mounted, then visualized and imaged using a fluorescence microscope (DM2500, Leica, Shanghai, China). Images were processed with ImageJ software (v.1.52).

### 4.8. Vector Construction

miR-143-3p mimics and corresponding negative controls (NC mimics) were synthesized by Shanghai Sangon Biotech Co., Ltd. (Shanghai, China). The miR-143-3p mimic sequence was 5′-UGAGAUGAAGCACUGUAGCUCG-3′, and the NC mimic sequence was 5′-UUGUACUACACAAAAGUACUG-3′. The *Itga6*-3′UTR sequence containing the miR-143-3p binding site was amplified by PCR using cDNA from goat SHFs with a pair of primers, *Itga6*-WT-UTR-F and *Itga6*-WT-UTR-R. Then, the amplified fragments were cloned in Dual-Luciferase miRNA Target Expression Vector (Promega, Madison, WI, USA) using *Pme* I and *Xba* I enzymes (NEB, Ipswich, MA, USA). The vectors were named *Itga6*-WT. Next, the *Itga6*-Mut vectors were generated with a mutagen in the miR-143-3p binding site by two pairs of mutagenic primers, *Itga6*-Mut-UTR-F and *Itga6*-Mut-UTR-R, using Mut Express II Fast Mutagenesis Kit V2 (Vazyme, Nanjing, China). All the primers used for vector construction are listed in Table S2.

### 4.9. Dual-Luciferase Assay

The empty plasmid was used as a blank control. The miR-143-3p mimic and NC mimic were co-transfected by Lipofectamine 2000 (Thermo Fisher, Waltham, MA, USA) into HEK293T cells with empty, *Itga6*-WT, or *Itga6*-Mut vectors when the cells were about 80% confluence in 96-well plates. After 48 h of co-culture, luciferase activity was examined using Dual-Luciferase Reporter Assays (Promega, Madison, WI, USA). The Infinite M200 Pro (TECAN, Männedorf, Switzerland) microplate reader detected the numerical size of luciferase, and the ratio of firefly luciferase values to Renilla luciferase values was calculated for analysis. Experiments were performed in triplicate.

### 4.10. Cell Culture and Transfection

The secondary hair follicle stem cells isolation processes followed the published protocol [43]. The secondary hair follicle stem cells of 2nd passage (P2) were seeded in 24-well plates to 80% confluence and the miR-143-3p mimic and NC mimic were transfected using Lipofectamine 2000 (Thermo Fisher, Waltham, MA, USA) according to the manufacturer’s instructions. At 48 h after transfection, cells were collected to detect the expression of miR-143-3p, *Itga6*, and *Ki67* by RT-qPCR.

### 4.11. Statistical Analysis

The *t* test was employed to analyze the significance of RT-qPCR data, and GraphPad Prism software (v.8.0.1) was used in the dual-luciferase reporter assay. A *p* value of <0.05 was considered statistically significant.

## 5. Conclusions

In summary, we discovered and characterized miRNA signatures from isolated SHFs. We demonstrated that many SHF miRNAs were expressed concordantly to function together, suggesting the markedly important roles of these miRNAs in regulating the SHF formation and cyclic growth. In addition, a functional analysis of predicted target genes clearly revealed the numerous signaling pathways involved in SHF regeneration and regression, including the ferroptosis pathway during anagen and catagen, which was not previously reported in the regression of hair follicles. We further revealed that miR-143-3p was preferentially expressed in SHFs and regulated the cashmere growth by repressing *Itga6* expression in Liaoning cashmere goats. Our identified miRNAs and pathways contribute to future research and provide a variety of promising targets for cashmere production.

## Data Availability

All results obtained in this study are available in the Supplementary Materials.

## Supplementary Materials

The supporting information can be downloaded at: https://www.mdpi.com/article/10.3390/ijms24021063/s1.
