# Supplementary material for: Discovery and Functional Analysis of Secondary Hair Follicle miRNAs during Annual Cashmere Growth
Source: Int J Mol Sci. 2023 Jan 5;24(2):1063. doi: 10.3390/ijms24021063 (PMC9864137; doi:10.3390/ijms24021063)
Supplement: Supplementary file 1 [file ijms-24-01063-s001.zip › Supplementary Files/SupplementaryLegends.pdf]

Supplementary Figure legends

Figure S1 Identification of miRNAs in SHFs. Proportion of known and novel miRNAs identified in SHFs.

Figure S2 KEGG pathway enrichment analysis of targeted genes of differentially expressed (DE) miRNAs between different phases in SHFs. The DE miRNAs were divided into four clusters revealed by hierarchical clustering. (A-C) Enriched KEGG pathways are shown for targeted genes of Cluster 1 miRNAs (A), Cluster 3 miRNAs (B) and Cluster 4 miRNAs (C). Rich factor indicates the ratio of target genes enriched in the pathway to genes annotated in the pathway.

Figure S3 Fluorescence in situ hybridization (FISH) assays of miR-143-3p in PHFs during anagen. The green signals represent the expression of miR-143-3p, the nuclei were stained with DAPI (blue). White arrowheads indicate the bulge region outlined by dashed line. Scale bars, 100 μm

Supplementary Table legends

Table S1 The mature sequences of predicted novel miRNAs and perfect match results of sequence alignment with five species in the miRbase database, including human (*Homo sapiens*), mouse (*Mus musculus*), cattle (*Bos taurus*), sheep (*Ovis aries*), and chicken (*Gallus gallus*).

Table S2 Primer sequences for miRNA and mRNA RT-qPCR analysis and *Itga6* vector construction
