# Supplementary material for: Discovery and Functional Analysis of Secondary Hair Follicle miRNAs during Annual Cashmere Growth
Source: Int J Mol Sci. 2023 Jan 5;24(2):1063. doi: 10.3390/ijms24021063 (PMC9864137; doi:10.3390/ijms24021063)
Supplement: Supplementary file 1 [file ijms-24-01063-s001.zip › Supplementary Files/Supplymentary Tables.pdf]

**Table S1 Information of novel miRNAs**

| Candidate Name | Mature Sequence | Perfect match in miRbase |
|---|---|---|
| chi-miR-203 | gugaaauguuuaggaccacuag | hsa-miR-203a-3p, mmu-miR-203-3p |
| novel_71 | aggcaagaugcuggcauagcugu | |
| novel_54 | cugugcgugugacagcggcuga | hsa-miR-210-3p, mmu-miR-210-3p |
| novel_587 | cuaagccagggauugugggu | |
| novel_98 | uugaacuguuaagaaccacugg | |
| novel_704 | auaucugugguuucugcac | |
| novel_100 | uguaacagcaacuccauguggaa | |
| novel_494 | uuuguucguucggcucgcguga | hsa-miR-375-3p, mmu-miR-375-3p |
| novel_120 | uagcagcgggaacaguacugcag | mmu-miR-503-5p |
| novel_24 | ugagguaguagguuguauaguu | hsa-let-7a-5p, mmu-let-7a-5p, bta-let-7a-5p, oar-let-7a |
| novel_130 | uaugugggacgguaaaccgcuu | mmu-miR-299a-3p |
| novel_697 | gaggguuuggguuuggucguggga | |
| novel_131 | uauugcacucgucccggccucc | hsa-miR-92b-3p, hsa-miR-92b-3p, bta-miR-92b |
| novel_148 | auagccaguuggggaagaaugc | bta-miR-7857-5p |
| novel_176 | agcugguguugugaaucaggccgu | |
| novel_463 | ugccaagcccacguucaaagg | bta-miR-6123 |
| novel_145 | uagguaguuucauguuguuggg | hsa-miR-196a-5p, mmu-miR-196a-5p, bta-miR-196a |
| novel_586 | aggaagucggagcugugcuuu | |
| novel_157 | aaaauccgaacgaacuuuuugg | |
| novel_182 | uaacagucuacagccauggucg | hsa-miR-132-3p, mmu-miR-132-3p, bta-miR-132 |
| novel_566 | ucucugucuucaauccuguagu | |
| novel_234 | agaaaccuggaugaacuuuuugg | bta-miR-2285r |
| novel_732 | uagaaucccacuccugacacca | |
| novel_218 | agcugguguugugaaucaggccg | hsa-miR-138-5p, mmu-miR-138-5p, bta-miR-138 |
| novel_206 | aguacuggagcuccacaugcaug | |
| novel_791 | auuauaggaauugaaccu | |
| novel_258 | aggccaaagcaaacgacugugc | |
| novel_498 | agaaaccgaacaaacuuuuuggc | |
| novel_421 | cucacacacacccucccccugcagu | |
| novel_306 | uaugugggacgguaaaccau | |
| novel_244 | gaaaguguaaggcaaggucugg | |
| novel_248 | aaaggauucugcugucggucccacu | hsa-miR-541-5p, oar-miR-541-5p |
| novel_241 | aacacccggacgaacuuuuugg | |
| novel_286 | aagaacccgagcgaacuuuugg | |
| novel_237 | agaguugagucuggacgucccg | hsa-miR-219a-1-3p, bta-miR-219 |
| novel_673 | aaucauacaaggacaauuucuuu | oar-miR-539-3p |
| novel_574 | ucacucagucgugcccgaauuacu | |
| novel_599 | uaaucucagcuggcaacugug | gga-miR-216a |

**Table S2 Primer sequences for miRNA and mRNA RT-qPCR and vector construction**

| miRNA Name | Forward primer sequence (5′ to 3′) | Reverse primer sequence (5′ to 3′) | RT stem-loop primer sequence (5′ to 3′) |
|---|---|---|---|
| chi-miR-143-3p | CCTGAGATGAAGCACTGT | GTCCGAGGTATTCGCACTGG | GTCGTATCCAGTGCAGGGTCCGAGGTATTCGCACTGGATACGACCGAGCT |
| chi-miR-203 | GGGGTGAAATGTTTAGGAC | CAGTGCGTGTCGTGGAGT | GTCGTATCCAGTGCAGGGTCCGAGGTATTCGCACTGGATACGACCGCCAATA |
| chi-miR-92a-3p | TGCCTATCGCACTTGTCC | CGCAGGGTCCGAGGTATTC | GTCGTATCCAGTGCAGGGTCCGAGGTATTCGCACTGGATACGACACAGGC |
| chi-miR-200a | TAACACTGTCTGGTAACGATGC | GCAGGGTCCGAGGTATTCGCACT | GTCGTATCCAGTGCAGGGTCCGAGGTATTCGCACTGGATACGACCATCGT |
| U6 | GCTTCGGCAGCACATATACTAAAAT | CGCTTCACGAATTTGCGTGTCAT | CGCTTCACGAATTTGCGTGTCAT |
| *Itga6* -WT-UTR | AGCTTTGTTTAAACGCCCTTAAGATATCAGTTGTGC | GCTCTAGATATCAAATGCAAACAGACCAG | |
| *Itga6* -MUT-UTR | AAATGCAGGAGGAAGTCAAGTCACTGGTCTGTTTGC | GACTTCCTCCTGCATTTTTAACAACTATAAACACCACTACATT | |
| *Itga6* | TCCATTACATTGCGTTTG | TCCACTATGATTGGCTCT | |
| *Ki67* | AAGAGGCAGCCCAAGTCAT | TTCTTAGCCTACTCCGGCCT | |
