# Supplementary figures and images for: Discovery and Functional Analysis of Secondary Hair Follicle miRNAs during Annual Cashmere Growth
Source: Int J Mol Sci. 2023 Jan 5;24(2):1063. doi: 10.3390/ijms24021063 (PMC9864137; doi:10.3390/ijms24021063)

**Figure S1**

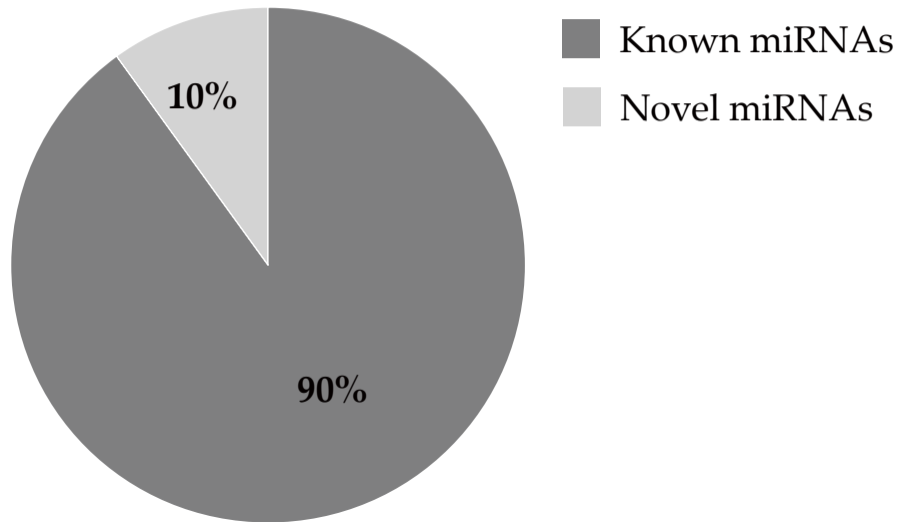

Figure S2

A

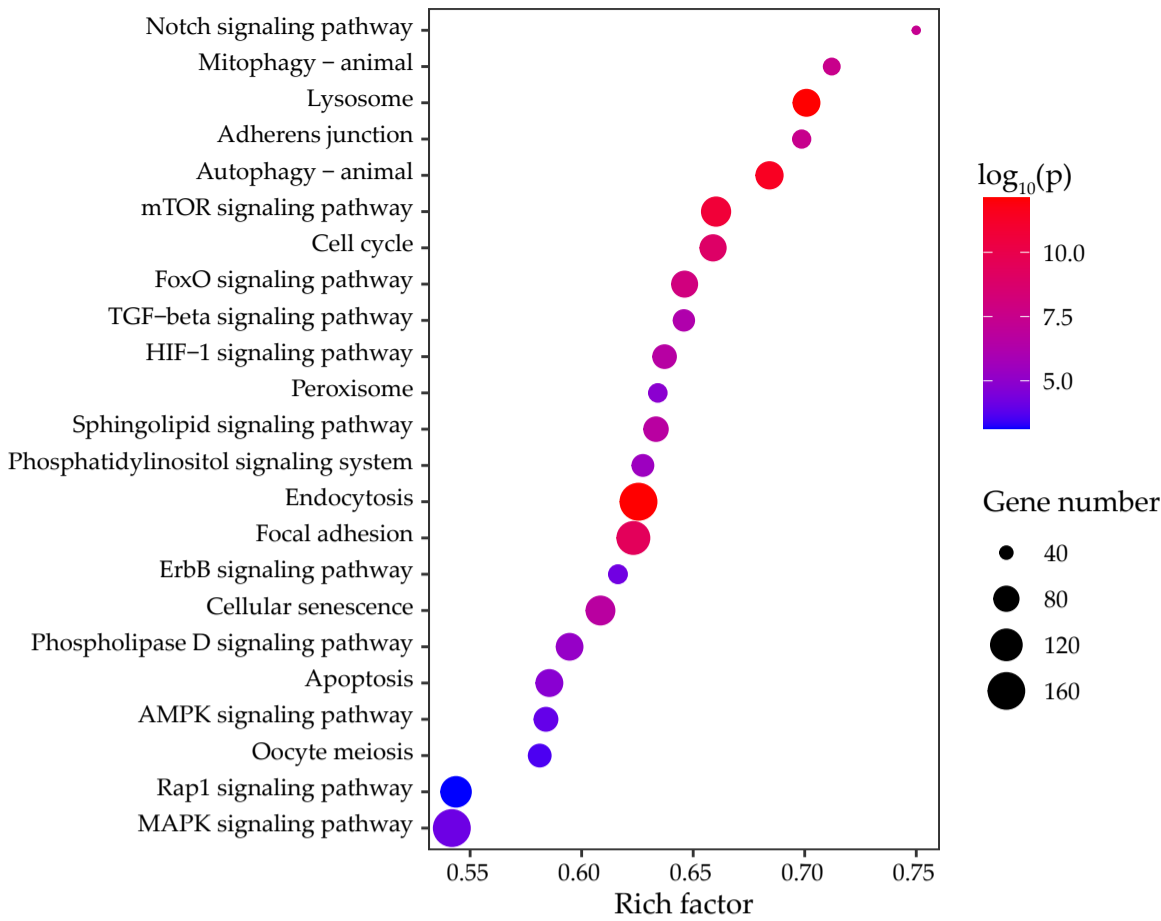

B

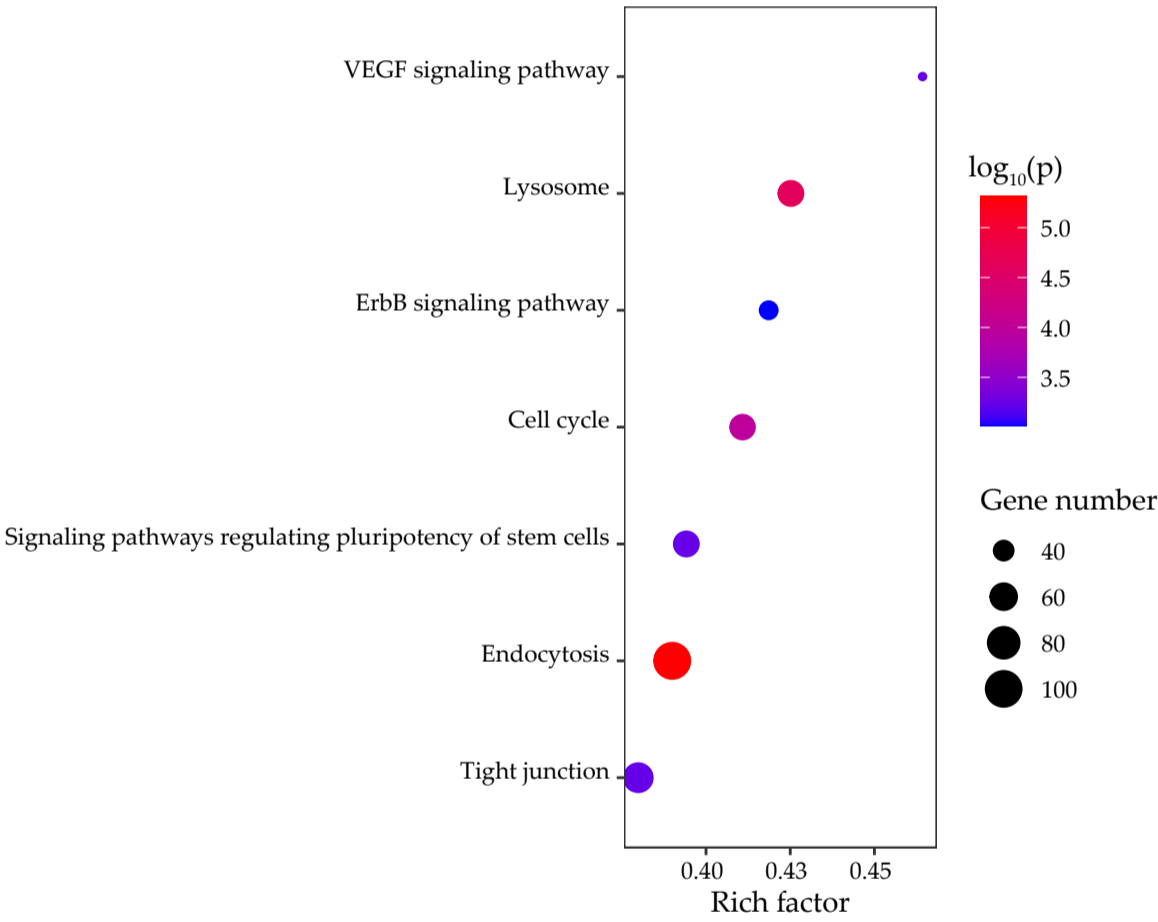

C

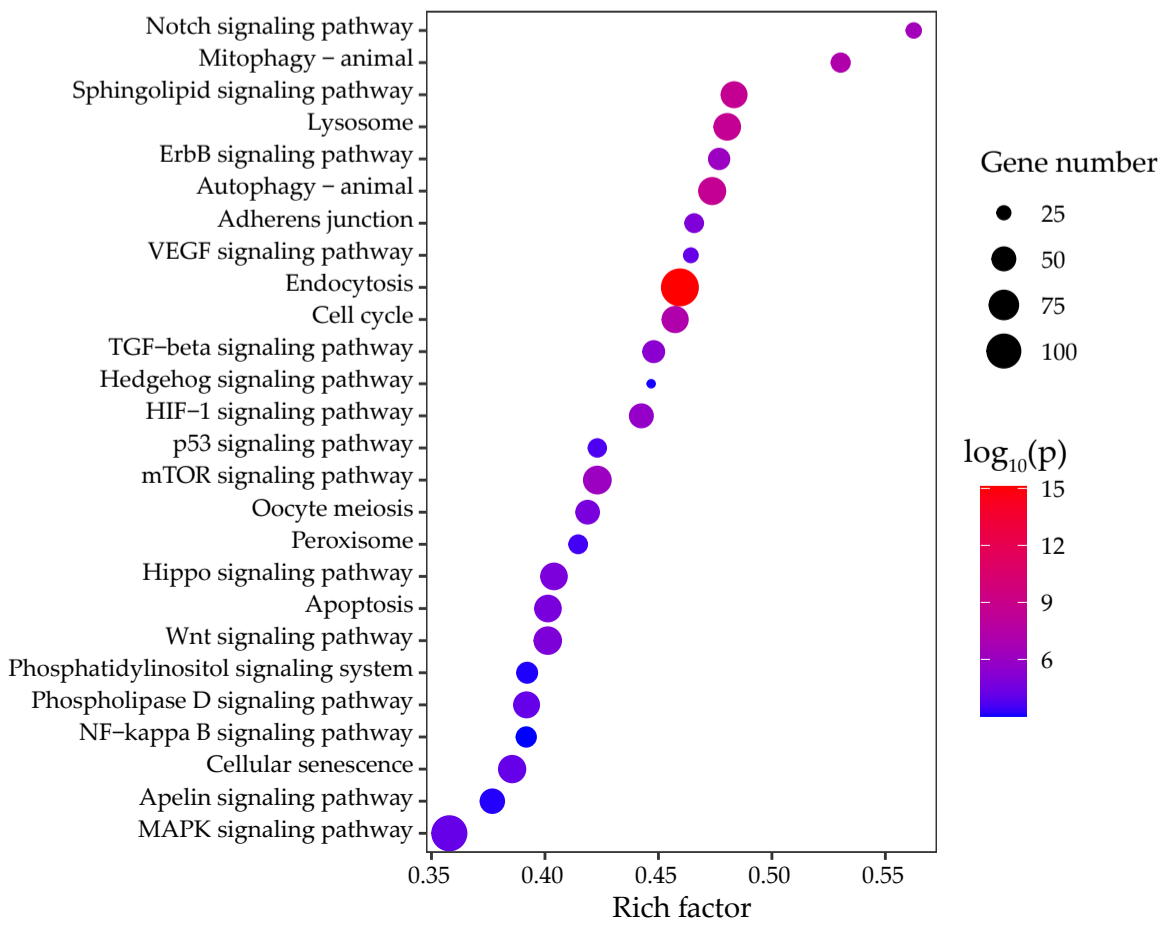

Figure S3

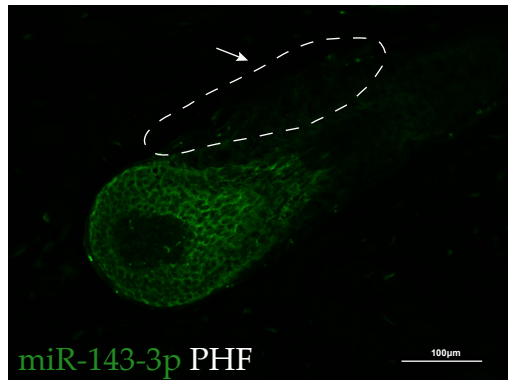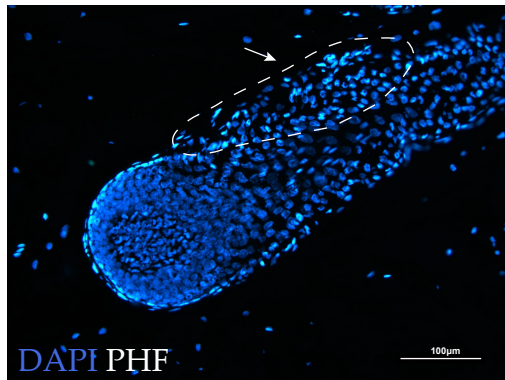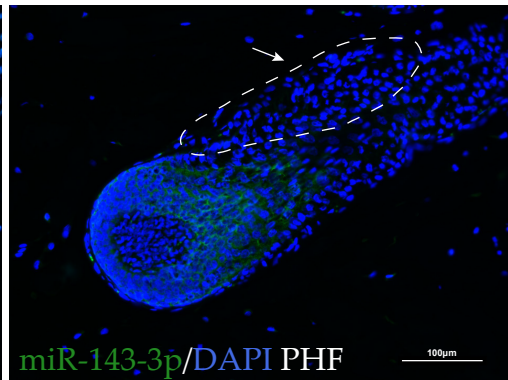

Supplement: Supplementary file 1 [file ijms-24-01063-s001.zip › Supplementary Files/Supplementary Figures.pdf]
